# A Load-Adaptive Driving Method for a Quasi-Continuous-Wave Laser Diode

**DOI:** 10.3390/mi15030355

**Published:** 2024-02-29

**Authors:** Yajun Wu, Wenqing Liu, Xinhui Sun, Jinxin Chen, Gang Cheng, Xi Chen, Yibin Fu, Pan Liu, Tianshu Zhang

**Affiliations:** 1 Anhui Institute of Optics and Fine Mechanics, Hefei Institutes of Physical Science, Chinese Academy of Sciences, Hefei 230031, China; yjwu@aiofm.ac.cn (Y.W.); wqliu@aiofm.ac.cn (W.L.); xhsun@mail.ustc.edu.cn (X.S.); jxchen@aiofm.ac.cn (J.C.); chenggang@mail.ustc.edu.cn (G.C.); cx0923@mail.ustc.edu.cn (X.C.); ybfu@aiofm.ac.cn (Y.F.); pliu@aiofm.ac.cn (P.L.); 2 Science Island Branch of Graduate School, University of Science and Technology of China, Hefei 230026, China; 3 Jiangsu Provincial Sensor Network Engineering Technology Research Center, Wuxi Institute of Technology, Wuxi 214121, China

**Keywords:** quasi-continuous wave, laser diode driver, pulsed constant-current source, load-adaptive driving method

## Abstract

A quasi-continuous-wave (QCW) laser diode (LD) driver is commonly used to drive diode bars and stacks designed specifically for QCW operations in solid-state lasers. Such drivers are optimized to deliver peak current and voltage pulses to LDs while maintaining low average power levels. As a result, they are widely used in laser processing devices and laser instruments. Traditional high-energy QCW LD drivers primarily use capacitors as energy storage components and pulsed constant-current sources with op-amps and power metal-oxide-semiconductor field-effect transistors (MOSFETs) as their core circuits for generating repeated constant-current pulses. The drawback of this type of driver is that the driver’s output voltage needs to be manually adjusted according to the operating voltage of the load before use to maximize driver efficiency while providing a sufficient current. Another drawback is its inability to automatically adjust the output voltage to maintain high efficiency when the load changes during the driver operation. Drastic changes in the load can cause the driver to fail to function properly in extreme cases. Based on the above traditional circuit structure, this study designed a stability compensation circuit and realized a QCW LD driver for driving a GS20 diode stack with a maximum repetition rate of 100 Hz, a constant current of approximately 300 A, a load voltage of approximately 10 V, and a pulse width of approximately 300 μs. In particular, a high-efficiency, load-adaptive driving method was used with the MOSFETs in the critical saturation region (i.e., between the linear and saturated regions), controlling its power loss effectively while achieving maximum output current of the driver. The experiments demonstrated that the driver efficiency could be maintained at more than 80% when the load current varied from 50 to 300 A.

## 1. Introduction

### 1.1. Description of the QCW LD Driver

Diode-pumped solid-state lasers (DPSSLs) are widely used in industrial, scientific, and military fields [1,2,3,4]. Traditionally, high-power pulsed DPSSLs have been obtained by Q-switching technology, typically using continuous-wave (CW) laser diode (LD) arrays to pump laser gain materials, such as Nd:YAG rods or slabs. However, pumping with QCW LD arrays yields more advantages. The peak power of a QCW LD array is higher than that of a CW array. In addition, a QCW pump can reduce the heat of a laser’s gain material and improve the quality of its laser beam, which is more desirable for users [5,6,7].

**Figure 1:**
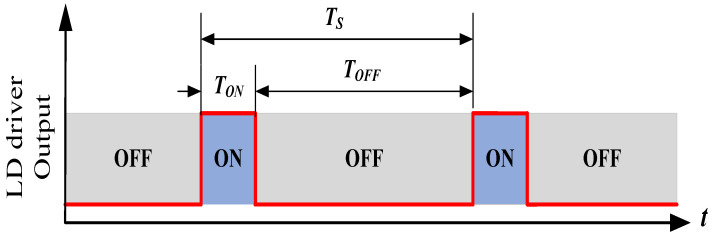
QCW LD driver output timing diagram.

A QCW LD driver is the pump source used to drive a QCW LD, bar, and stack, and it is switched on only for time intervals short enough to reduce thermal effects significantly but long enough to bring the laser process close to its steady state, as shown in Figure 1. In other words, the laser is optically in the state of CW operation. The duty cycle may be only a few percent, thus greatly reducing heating and all related thermal effects, such as thermal lensing and damage through overheating [8,9,10]. To maintain consistent power of each laser pulse, the driver typically requires a constant-current output [11,12,13,14]. Such drivers are designed to deliver peak current pulses to LDs while maintaining low average power levels. The duty cycle *D* of such a pulse current is calculated in Equation (1):

(1) $$D=\frac{T_{ON}}{T_{S}}$$

where $T_{ON}$ is the current pulse *ON* state duration and $T_{S}$ is the cycle duration. In Figure 1, $T_{OFF}$ is the current pulse OFF state duration, and *t* is the timeline.

### 1.2. Principle and Characteristics of a Conventional QCW LD Driver

In general, a switching mode power supply (SMPS)-based LD driver is more popular than a linear driver because of its high efficiency and compact size. Sharma et al. proposed a QCW LD driver based on a high-frequency SMPS capable of operating at a repetition rate of 100 Hz and providing a maximum pulse current of ∼40 A to drive an LD operating at ∼3 V, with rising/falling times of up to 5 ms [15]. Later, they aimed to increase the output voltage and current to 4 V and 50 A, respectively, and reduced the current rising/falling time to ∼80 μs [16]. However, QCW LD drivers with fast rising/falling times, high voltages, and high current outputs are challenging to implement using only SMPS techniques, and the relevant literature is quite limited. The common practice is to split a QCW LD driver into two parts [17,18,19]. The first part is the SMPS, which provides the AC-to-DC voltage conversion, and to accumulate sufficient energy before generating a pulse current, a capacitor bank of tens to hundreds of microfarad is essential. The other part is a pulsed constant-current source that uses MOSFETs, bipolar junction transistors, and other power components as linear regulators for current regulation. To increase the current output capacity, using multiple constant-current circuits in parallel is an efficient option [20,21,22,23].

**Figure 2:**
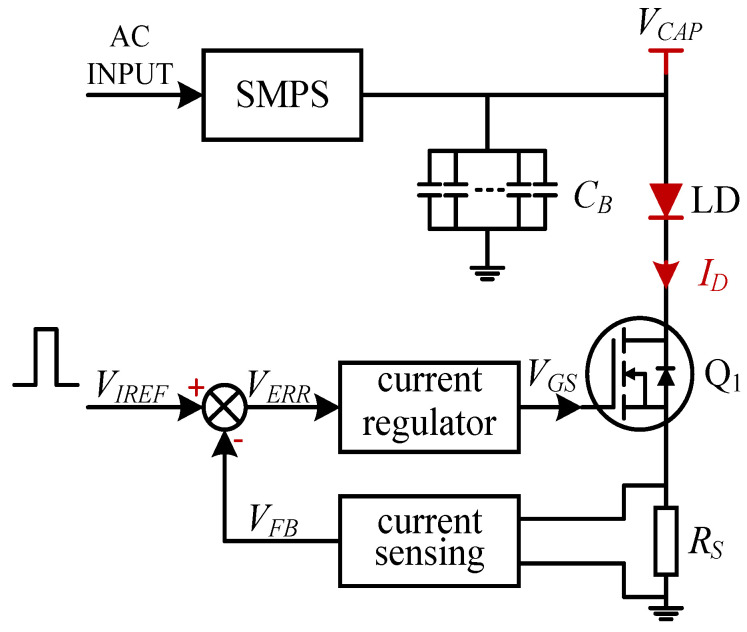
Schematic of a typical simplified QCW LD driver.

Figure 2 shows a schematic of a typical simplified QCW LD driver. The AC voltage is passed through the SMPS to generate a constant DC voltage $V_{CAP}$ suitable for the system’s operation. In general, this voltage is required so that the MOSFET operates in the saturation region to attain a sufficient voltage margin against possible fluctuations. To provide sufficient power at discharge, a capacitor bank needs to be placed at the SMPS output. The current flowing through the LD is controlled using a closed-loop feedback circuit with the input voltage $V_{IREF}$ as a reference. Specifically, the output current $I_{D}$ flowing through the LD is sampled by the sampling resistance $R_{S}$ and passed through the current-sensing module to become the feedback voltage $V_{FB}$, which is compared with the reference voltage $V_{IREF}$ to obtain the error signal $V_{ERR}$. After passing through the current regulator, the gate-to-source voltage $V_{GS}$ of the MOSFET $Q_{1}$ is generated to control the current $I_{D}$, which is represented by Equation (2), where $A_{FB}$ is the gain of the feedback circuit:

(2) $$I_{D}=\frac{V_{IREF}}{A_{FB}\times R_{S}}$$

During the discharge period of the capacitor bank, the voltage decreases. We can temporarily ignore the charging current, assuming the output current $I_{D}$ is much larger, and the voltage drop of the capacitor bank can be obtained using Equations (3) and (4):

(3) $$Q=C_{B}\times \Delta U=I_{D}\times T_{ON}$$

(4) $$\Delta U=\frac{I_{D}\times T_{ON}}{C_{B}},$$

where *Q* is the total charge, $C_{B}$ is the total capacitance of the capacitor bank, ΔU is the voltage drop of the capacitor bank.

The minimum voltage $V_{CAP}$ required by the capacitor bank, a difficult parameter to determine, is calculated in Equation (5). For an LD driver user, although $V_{LD}$ can be found in the supplier manual, it shifts with the load, and $R_{S}$ and $C_{B}$ are known only to the designer and not usually defined in the manual. Furthermore, designers find evaluating the optimal parameter $V_{DS}$ difficult. As mentioned, a MOSFET operating in a saturation region can better regulate $I_{D}$ via gate voltages and ensure a sufficient voltage margin. According to MOSFET characteristics, the drain-to-source voltage satisfying this condition can be expressed by Equation (6). Unfortunately, $V_{TH}$ is related to not only the fabrication process of the chip but also the operating temperature, and thus, the minimum $V_{DS}$ is not fixed. Therefore, users are often forced to set a sufficiently large $V_{CAP}$ in case of a system failure at the expense of power efficiency.

(5) $$V_{CAP}=V_{LD}+V_{DS}+R_{S}\times I_{D}+\Delta U=V_{LD}+V_{DS}+\left(R_{S}+\frac{T_{ON}}{C_{B}}\right)\times I_{D}$$

(6) $$V_{DS}\geq V_{GS}-V_{TH}$$

where $V_{LD}$ is the operating voltage of the *LD*, $V_{DS}$ is the drain-to-source voltage of the MOSFET, $V_{TH}$ is the gate threshold voltage of the MOSFET, and $R_{S}$ is the sampling resistance.

When the *LD* is working in a constant-current state, it is approximately equivalent to a small resistor with resistance $R_{LD}$, and $V_{LD}$ can be represented by Equation (7) [22,24]:

(7) $$V_{LD}\approx V_{LD\_TH}+R_{LD}\times I_{D}$$

We can then substitute Equation (7) into Equation (5):

(8) $$\begin{array}{cc} V_{CAP} & =\left(V_{LD\_TH}+R_{LD}\times I_{D}\right)+V_{DS}+\left(R_{S}+\frac{T_{ON}}{C_{B}}\right)\times I_{D}\\ \; & =V_{LD\_TH}+V_{DS}+\left(R_{LD}+R_{S}+\frac{T_{ON}}{C_{B}}\right)\times I_{D} \end{array}$$

where $V_{LD\_TH}$ is the threshold voltage of the *LD* and $R_{LD}$ is the equivalent resistance of the *LD*.

In Equation (8), $V_{LD\_TH}$, $R_{LD}$, $R_{S}$ and $C_{B}$ are treated as fixed values. If the user sets the voltage $V_{CAP}$ of the SMPS to a fixed value, then the $V_{DS}$ decreases/increases as $T_{ON}$ and $I_{D}$ increase/decrease, but $V_{DS}$ shifts more significantly with $I_{D}$.

From the above analysis, we see that regular LD drivers suffer from the following shortcomings:

(1) Before using a driver, the user should estimate the minimum voltage $V_{CAP}$ of the SMPS according to the load and the characteristics of the driver (refer to Equation (5)). Specifying values for $R_{S}$, $C_{B}$ and $V_{DS}$ is challenging, and so, a perfect $V_{CAP}$ is also difficult to obtain, usually only possible through complex experiments to estimate a median value, potentially sacrificing power efficiency by leaving too great a margin.
(2) In the case of a fixed $V_{CAP}$, $V_{DS}$ decreases with increasing loads, the MOSFET may enter the linear region if $V_{CAP}$ does not have sufficient margin, and, in extreme cases, $I_{D}$ does not reach the set value. In addition, $V_{DS}$ increases as the load decreases, and then the MOSFET losses increase, reducing the efficiency of the power supply.

### 1.3. Principle and Advantages of Load-Adaptive QCW LD Drivers

#### 1.3.1. Features of MOSFETs

**Figure 3:**
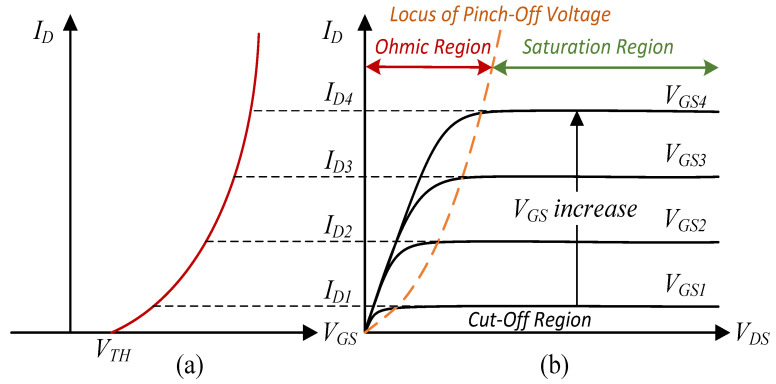
N-channel-enhancement-type MOSFET: (**a**) transfer and (**b**) output characteristics.

Before describing the load-adaptive LD driver principle, we need to understand the characteristics of power MOSFETs. MOSFETs can be thought of as voltage-controlled current switches. In general, MOSFETs exhibit three operating regions, as shown in Figure 3 with the n-channel power MOSFETs as an example [25], as follows:

 *(1)*  *Cut-off region*

A cut-off region is a region in which a MOSFET is OFF, as no current is flowing through it, which can be expressed by Equations (9) and (10):

(9) $$V_{GS}<V_{TH}$$

(10) $$I_{D}=0$$

 *(2)*  *Ohmic or linear region*

An ohmic, or linear, region is a region where the current $I_{D}$ increases with $V_{DS}$, as indicated by Equations (11)–(13):

(11) $$V_{DS}<V_{GS}-V_{TH}>0$$

(12) $$I_{D}=\frac{1}{2}\mu_{n}C_{ox}\frac{W}{L}\left[2\left(V_{GS}-V_{TH}\right)V_{DS}-V_{DS}^{2}\right]$$

(13) $$g_{m}=\frac{\partial }{\partial V_{GS}}\left\{\frac{1}{2}\mu_{n}C_{ox}\frac{W}{L}\left[2\left(V_{GS}-V_{TH}\right)V_{DS}-V_{DS}^{2}\right]\right\}=\mu_{n}C_{ox}\frac{W}{L}V_{DS},$$

where $\mu_{n}$ is the channel mobility, $C_{ox}$ is the oxide capacitance density, *W* is the channel width, *L* is the channel length, and $g_{m}$ is the transconductance.

 *(3)*  *Saturation region*

In a saturation region, the MOSFET $I_{D}$ remains constant despite increases in $V_{DS}$ and occurs once $V_{DS}$ exceeds the pinch-off voltage $V_{GS}-V_{TH}$, as expressed by Equations (14)–(16), as follows:

(14) $$V_{DS}\geq V_{GS}-V_{TH}>0$$

(15) $$I_{D}=\frac{1}{2}\mu_{n}C_{ox}\frac{W}{L}{\left(V_{GS}-V_{TH}\right)}^{2}$$

(16) $$g_{m}=\frac{\partial }{\partial V_{GS}}\left\{\frac{1}{2}\mu_{n}C_{ox}\frac{W}{L}{\left(V_{GS}-V_{TH}\right)}^{2}\right\}=\mu_{n}C_{ox}\frac{W}{L}\left(V_{GS}-V_{TH}\right)$$

Additionally, $I_{D}$ reaches its maximum value when $V_{DS}=V_{GS}-V_{TH}$. Afterward, although $V_{DS}$ increases, $I_{D}$ remains nearly constant, but the thermal power consumption of the MOSFET increases.

From the above description, we easily see two ways to tune the output current of a MOSFET: adjusting $V_{DS}$ in the linear region and adjusting $V_{GS}$ in the saturated region. The latter is a common approach for fast current control. However, an appropriate $V_{DS}$ for different load currents is necessary, as discussed below.

#### 1.3.2. Load-Adaptive Driving Method

It is worth noting that Reference [26] describes a method for controlling the maximum efficiency output of a voltage-controlled adjustable constant-current source:

(17) $$V_{CAP}=I_{D}\left(R_{load}+R_{S}+R_{on}\right)$$

Here, $R_{load}$ represents the load resistance value, which denotes the equivalent resistance of the LD, and $R_{on}$ is the MOSFET internal resistance. The value of $V_{CAP}$ can be calculated if the above parameters are known. Unfortunately, $R_{load}$ and $R_{on}$ are generally uncertain in practical applications, although reference values maybe provided in datasheets. These values vary with manufacturing process and operating temperature. Existing techniques can only estimate these parameters through experiments, and the results of estimation are not accurate. Additionally, the MOSFET operates in the Ohmic region, and the output characteristics are sensitive to voltage fluctuations of $V_{DS}$. The severe fluctuations in $V_{DS}$ may lead to unstable output. In summary, the method in [26] cannot be adopted in this study because of these reasons above. Instead, the operating point of the MOSFET is set to the critical saturation region to optimize efficiency while ensuring stable operation of the driver.

**Figure 4:**
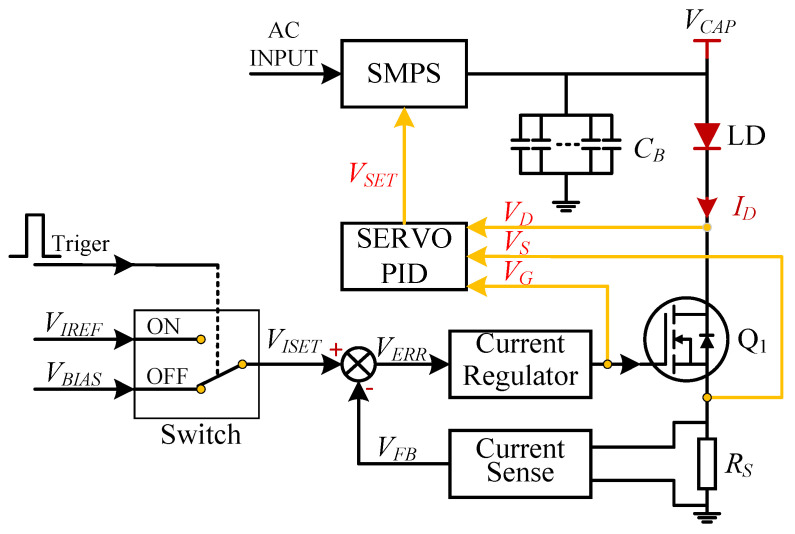
Block diagram of the load-adaptive QCW LD driving scheme.

**Figure 5:**
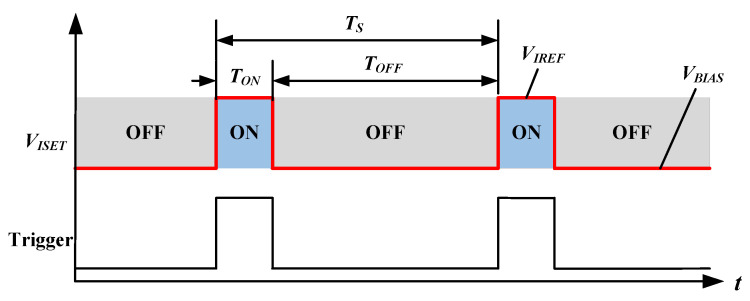
Switching waveform of the reference voltage for the closed-loop feedback circuit.

**Figure 6:**
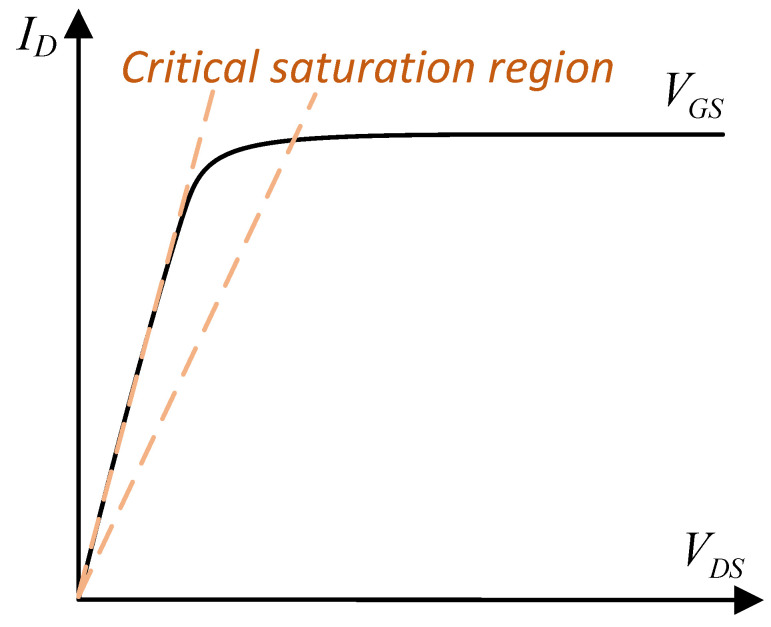
Critical saturation region of a MOSFET.

Based on the characteristics of a MOSFET, we propose an efficient, load-adaptive QCW LD driving scheme, as shown in Figure 4. An analog switch is used to switch the reference voltage $V_{ISET}$ of the closed-loop feedback circuit between $V_{IREF}$ and $V_{BIAS}$ via the trigger, and the control waveform is shown in Figure 5. A servo controller detects $V_{G}$, $V_{D}$ and $V_{S}$, and the output voltage $V_{CAP}$ of the SMPS is tuned by the servo PID such that the MOSFET operates in the critical saturation region, as illustrated in Figure 6. This approach uses a PID controller to automatically lock $V_{DS}$ at a small and safe operating voltage, avoiding the hassle and risk of manually setting $V_{CAP}$ and improving power efficiency.

The specific workflow of the servo controller is as follows:

(1) Choose the operating point in the critical saturation region.

According to the characteristics of a MOSFET, the transconductance $g_{m}$ reaches its maximum value at the critical saturation operating point, where $V_{DS}=V_{GS}-V_{TH}$. If we need to reduce MOSFET losses further or focus on reliability, $V_{DS}$ can be multiplied by a factor, as shown in Equation (18). When a<1, the MOSFET losses are smaller; when a>1, the resistance-to-power perturbations improve. Of note, *a* is close to unity for the sake of both efficiency and stability. In this study, a=1:

(18) $$V_{DS\_SET}=a\left(V_{GS}-V_{TH}\right),a>0$$

(2) Obtain the threshold voltage $V_{TH}$

As discussed earlier, $V_{TH}$ is affected by factors such as the chip fabrication process and temperature, and it is not a fixed value. We set $V_{BIAS}$ to a small voltage such that the MOSFET has an extremely small current $I_{D}$ when the MOSFET is OFF. The gate voltage $V_{G}$ at this time can be approximated as the threshold voltage $V_{TH}$, which can be measured at each driving cycle.

(3) Control $V_{DS}$ automatically

**Figure 7:**
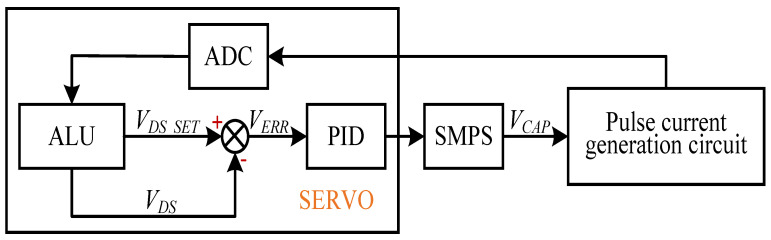
Block diagram of the automatic control of $V_{DS}$.

We lock $V_{DS}$ indirectly at the critical saturation operating point $V_{DS\_SET}$ by controlling $V_{CAP}$. Figure 7 shows a simplified control block diagram. After the LD driver engages, the servo controller samples $V_{G}$, $V_{D}$, and $V_{S}$ at the ON state of each cycle by ADCs (analog to digital converters) and calculates $V_{DS}$ and $V_{DS\_SET}$ using an ALU (arithmetic logic unit). The reference voltage $V_{DS\_SET}$ is calculated using Equation (18), and the error $V_{ERR}$ is obtained using Equation (19). The PID controls the voltage $V_{CAP}$ of the SMPS in the subsequent OFF state, such that the error $V_{ERR}$ tends toward zero:

(19) $$V_{ERR}=V_{DS}-\left(V_{GS}-V_{TH}\right)=V_{D}-V_{G}+V_{TH}$$

#### 1.3.3. Advantages of Load-Adaptive QCW LD Drivers

The advantages of adopting a load-adaptive QCW LD driver as described above are summarized below:

(1) Traditional drivers require users to estimate the SMPS output voltage according to the driver and load parameters, or the user must estimate the appropriate $V_{CAP}$ through complex experiments, which are difficult and troublesome. Thus, either the power efficiency is low or the ability to resist voltage disturbance is poor. Our driver can automatically find the optimal operating voltage, thereby reducing the need for manual manipulation and its associated risks.
(2) Traditional drivers typically operate a MOSFET in the saturation region, where $V_{CAP}$ has a large margin, resulting in large $V_{DS}$ and MOSFET losses. Our driver enables the MOSFET to operate in the critical saturation region, minimizing $V_{DS}$ and, thus, MOSFET losses and improving power efficiency.
(3) Traditional drivers have a fixed $V_{CAP}$ during operation and cannot adapt to load changes automatically. Our driver can detect the operating voltage of the three terminals of the MOSFET in real time and recalculate the appropriate operating voltage $V_{DS}$ when the load changes, such that the MOSFET can keep working in the critical saturation region and maintains high power efficiency.

## 2. Design and Implementations

### 2.1. Design Specifications and Block Diagrams

**Figure 8:**
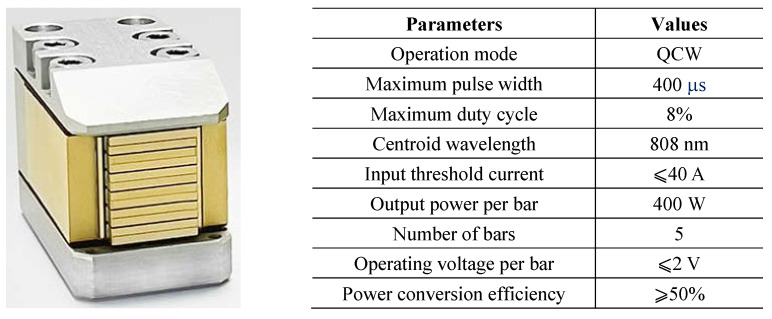
GS20 LD stack with its main parameters.

**Figure 9:**
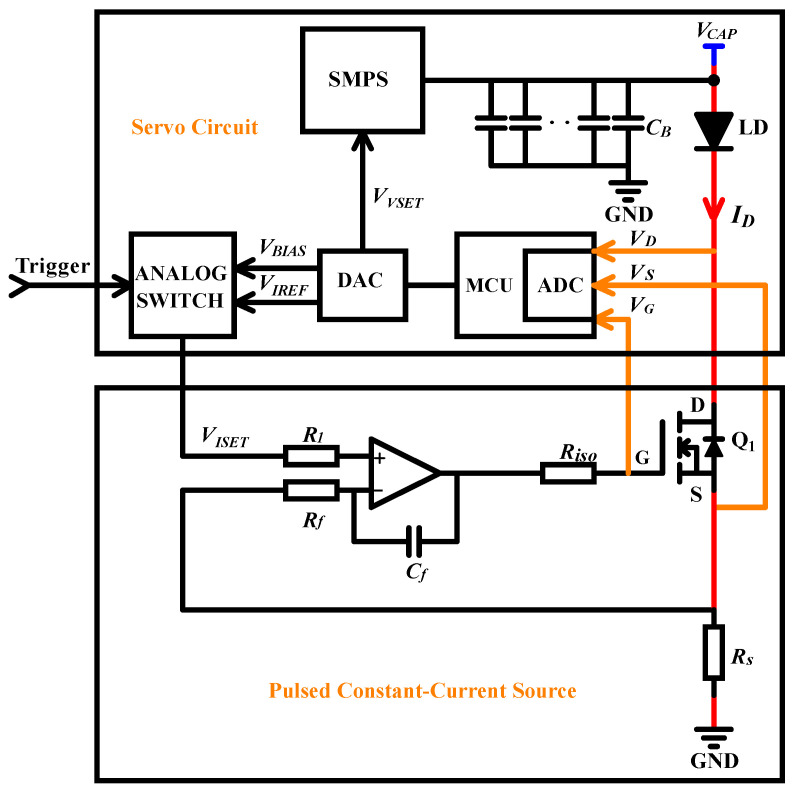
Design block diagram of the QCW LD driver.

As shown in Figure 8, a high-efficiency QCW LD driver is required in our laser system to drive the laser stack. We designed the circuit based on the parameters in Table 1, and a diagram of the circuit block is shown in Figure 9. The driver comprises a servo circuit and a pulsed constant-current source. The latter improves the power supply capacity through a multistage parallel operation [21,22,23]. Because of the development of high-power MOSFETs, we meet the design requirements with a single module and a simplified circuit structure.

### 2.2. Selection of the Main Components

#### 2.2.1. Selection of a MOSFET

The MOSFET is the most critical component of a driver, and we chose the IXYS IXTH360N055TT2, with a maximum drain-to-source current of 360 A, to meet our requirements. The MOSFET’s parameters are listed in Table 2.

#### 2.2.2. Selection of the Op-Amps

Op-amps are used in the constant-current source circuit. We use TI’s OPA2197, with a 10 MHz bandwidth and an extremely low input offset voltage and input offset current. Its parameters are listed in Table 3.

#### 2.2.3. Capacity of the Capacitor Bank

The driver uses a capacitor bank for energy storage, and $V_{CAP}$ drops by ΔU during discharge, but the current output capacity of the driver is not affected. We calculate the capacity of the capacitor bank using Equations (20) and (21). According to Table 1, $I_{D}$ and $T_{ON}$ are 300 A and 300 μs, respectively. The effect on the power efficiency is reduced by setting ΔU to 1 V. The calculated $C_{B}$ must be at least $9\times 10^{4}$μF, and we use $12\times 10^{4}$μF, preserving a 25% margin:

(20) $$I_{D}\times T_{ON}=C_{B}\times \Delta U$$

(21) $$C_{B}=\frac{I_{D}\times T_{ON}}{\Delta U}=\frac{300\times 300\times 10^{-6}}{1}=9\times 10^{4}\;\mu F$$

#### 2.2.4. Selection of the SMPS

When selecting an SMPS, the primary consideration is the power supply capacity, which is calculated using Equations (22) and (23):

(22) $$I_{SMPS}\times T_{S}=I_{D}\times T_{ON}$$

(23) $$I_{SMPS}=\frac{I_{D}\times T_{ON}}{T_{S}}=\frac{300\times 300\;\mu s}{10,000\;\mu s}=9\;A$$

where $I_{D}$ is the maximum output current of the LD driver (i.e., 300 A), $T_{ON}$ is the pulse width (i.e., 300 μs), $T_{S}$ is the pulse period (i.e., 10 ms), and $I_{SMPS}$ is the minimum output current of the SMPS (i.e., 9 A).

A Cotek AE-1500-24 was selected as the SMPS to allow for a sufficient power margin and subsequent product upgrades. Its main parameters are listed in Table 4.

#### 2.2.5. Selection of the Sampling Resistor

The precision resistor WSLP40261L000FEA with a resistance of 1 mΩ and a power rating of 7 W is used to sample the current. The peak power $P_{R_{S}}$, average power $\bar{P_{R_{S}}}$, and operating voltage $V_{RS}$ of the resistor are calculated using Equations (24)–(26), respectively:

(24) $$P_{R_{S}}=I_{D}^{2}\times R_{S}=300^{2}\times 10^{-3}=90\;W$$

(25) $$\bar{P_{R_{S}}}=P_{R_{S}}\times D=90\times 3\%=2.7\;W$$

(26) $$V_{RS}=I_{D}\times R_{S}=0.3\;V$$

where $R_{S}$ is the sampling resistance of 1 mΩ and *D* is the duty cycle of $\frac{300\;\mu s}{10\;\mathrm{ms}}=3\%$.

### 2.3. Pulsed Constant-Current Source Circuit

#### 2.3.1. Stability Analysis

**Figure 10:**
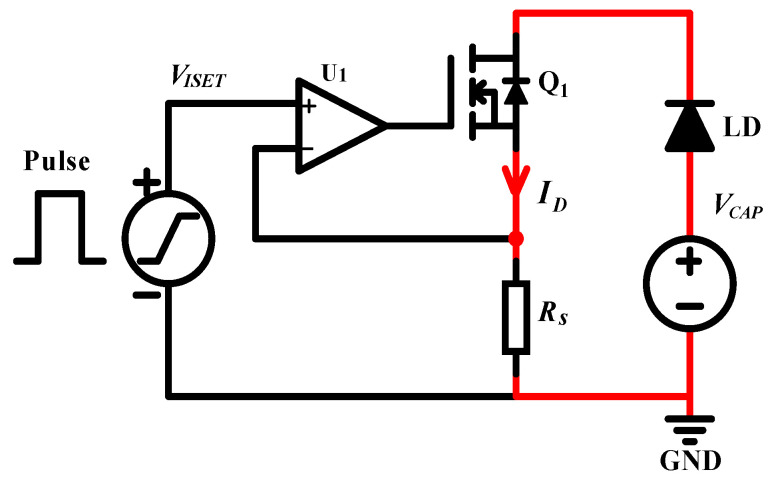
Basic model of the QCW LD driver based on the MOSFET.

**Figure 11:**
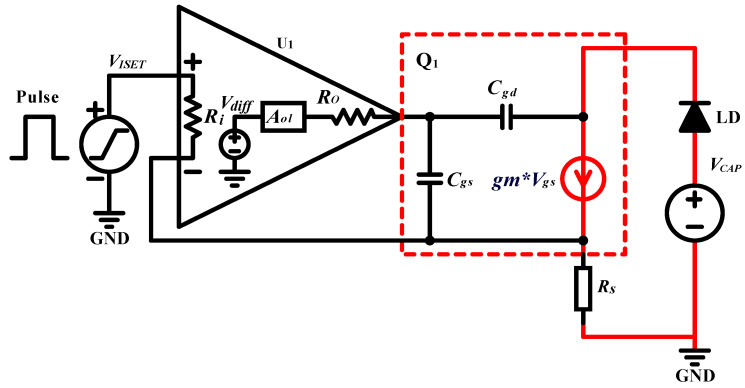
High-frequency, small-signal model of the QCW LD driver.

A QCW LD driver, which uses a MOSFET-based transconductance amplifier as the key constant-current circuit [27,28], is essentially a voltage-controlled constant-current source, as shown in Figure 10. Since a pulsed constant-current source is needed, the stability of the circuit is a key consideration. To further analyze the stability of the circuit, its high-frequency and small-signal equivalent model is considered, as shown in Figure 11. The input resistance $R_{i}$ of the op-amp $U_{1}$, which is typically very large, can be considered an open circuit. The input differential voltage $V_{diff}$ is approximately equal to $V_{ISET}$, $A_{ol}$ is the open-loop gain, and $R_{o}$ is the open-loop output impedance. According to the data sheet, $A_{ol}$ is ∼134 dB, $R_{o}$ is ∼375 is ohms, and the unity gain bandwidth is ∼10 MHz. For power MOSFETs, the parasitic capacitances, such as $C_{gs}$ and $C_{gd}$, are typically very high. According to the IXTH360N055T2 data sheet, $C_{iss}=C_{gs}+C_{gd}\approx 20$ nF was measured at 1 MHz, with a gate-to-source voltage of 0 V and a drain-to-source voltage of 25 V. Thus, the effects of $C_{gs}$ and $C_{gd}$ cannot be neglected. The open-loop gain curve of an op-amp typically has a low-frequency pole; according to the OPA2197 manual, $f_{P1}\approx 2$ Hz. A second pole introduced in the open-loop gain curve is due to the effect of the open-loop output resistance $R_{o}$ and the MOSFET input capacitance $C_{iss}$. Here, we neglect the effects of $R_{s}$ and $R_{LD}$ since their values are much smaller than $R_{o}$. The frequency of the second pole, $f_{P2}$, is calculated using Equation (27):

(27) $$f_{P2}=\frac{1}{2\pi R_{o}C_{iss}}=\frac{1}{2\pi \times 375\times 20\times 10^{-9}}\approx 21.23\;\mathrm{kHz}$$

**Figure 12:**
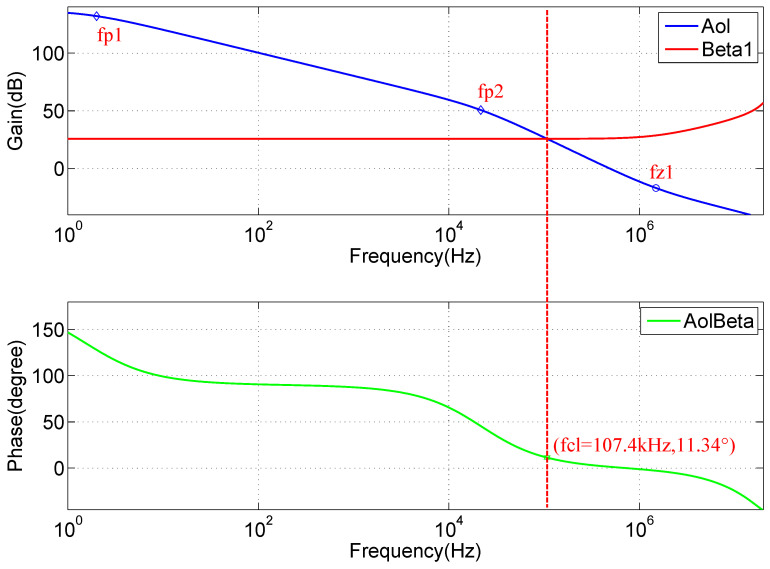
SPICE model simulation curves of the QCW LD driver circuit.

The SPICE model of the circuit was used for the simulations, and the resulting curves are shown in Figure 12. The position of the second pole of the $A_{ol}$ curve is $f_{P2}=21.62$ kHz, which is very close to our estimated value. The open-loop gain curve $A_{ol}$ intersects the closed-loop gain curve *Beta1* (1/β ) at a frequency of $f_{CL}=107.40$ kHz, where the rate of closure is approximately −40 dB/decade. The loop gain curve, *AolBeta*, has a phase margin of $11.34^{\circ }$ at this point, indicating that the circuit is unstable. We also find that $A_{ol}$ is zero at $f_{Z1}=1496.24$ kHz, which is caused by parasitic parameters that are not considered, but this does not affect the stability analysis.

#### 2.3.2. Compensation

**Figure 13:**
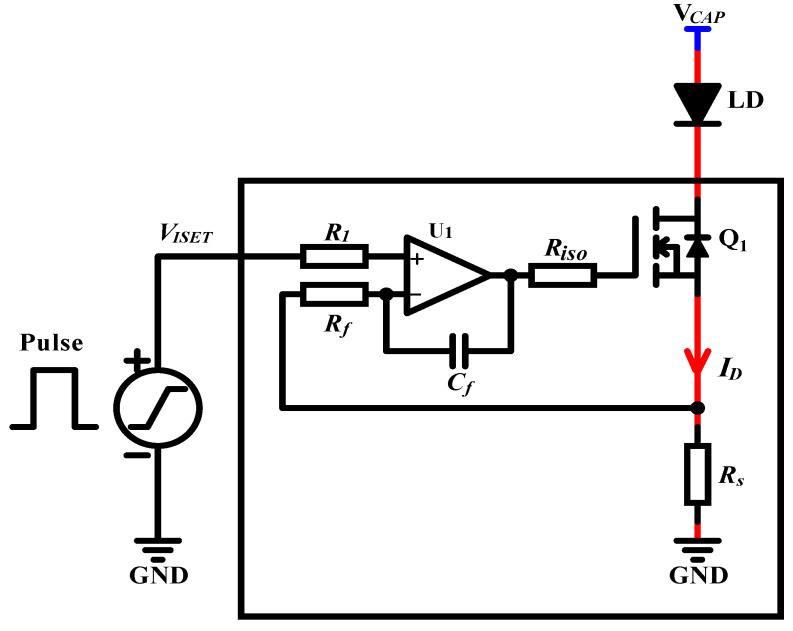
Pulsed constant-current source circuit with compensation.

The above analysis shows that the pulsed constant-current source circuit is unstable because of the parasitic capacitance of the MOSFET. This study adopts the method used in [29] for circuit compensation but includes the SPICE simulation description of the improved circuit. However, a comprehensive stability analysis is beyond the scope of this study [30]. The compensation circuit is shown in Figure 13. First, a resistor, $R_{iso}$, placed between the op-amp output and the gate terminal of the MOSFET helps isolate the op-amp from the capacitive load. Second, the feedback capacitor, $C_{f}$, provides feedback directly from the output of the op-amp to the inverting input, removing the MOSFET gain from the high-frequency feedback loop and replacing it with DC feedback from the MOSFET source terminal via the feedback resistor $R_{f}$. If these three components are not used, the circuit is unstable. In general, the compensation component of such a circuit is not set by a fixed equation but rather by choosing values and then manipulating them while observing the output response. The values selected during circuit simulation are $R_{iso}=5.1$ Ω, $R_{f}=1$ kΩ, and $C_{f}=470$ pF.

#### 2.3.3. Effect of Compensation on the $A_{ol}$ Curve

With resistor $R_{iso}$, the position of the second pole, $f_{P2}$, which is generated on the $A_{ol}$ curve, is shifted slightly to a lower frequency. Moreover, introducing a zero $f_{Z2}$ at a higher frequency increases the slope of the $A_{ol}$ curve by 20 dB/decade after this point, which helps stabilize the circuit. The values of $f_{P2}$ and $f_{Z2}$ are calculated using Equations (28) and (29):

(28) $$f_{P2}=\frac{1}{2\pi \left(R_{O}+R_{iso}\right)C_{iss}}\approx \frac{1}{6.28\times 380.1\times 20\times 10^{-9}}\approx 20.95\;\mathrm{kHz}$$

(29) $$f_{Z2}=\frac{1}{2\pi R_{iso}C_{iss}}\approx \frac{1}{6.28\times 5.1\times 20\times 10^{-9}}\approx 1561.13\;\mathrm{kHz}$$

#### 2.3.4. Effect of Compensation on the Beta1 Curve

Before studying the Beta1 curve, Figure 12 shows the simulation results, where, at the low-frequency region below $f_{CL}$, the closed-loop gain is largely unchanged, as expressed by Equation (30). In addition, β is helpful for our next calculation:

(30) $$\frac{1}{\beta }\approx 25.74\;\mathrm{dB}\approx 19.36$$

After adding $C_{f}$ and $R_{f}$, a zero-value $f_{Z3}$ and pole $f_{P3}$ are generated on the Beta1 curve, and their positions are calculated using Equations (31) and (32), respectively:

(31) $$f_{Z3}=\frac{1}{2\pi R_{f}C_{f}}=\frac{1}{2\pi \times 10^{3}\times 470\times 10^{-12}}\approx 338.80\;\mathrm{kHz}$$

(32) $$f_{P3}=\frac{\beta }{2\pi R_{f}C_{f}}=\frac{\frac{1}{19.36}}{2\pi \times 10^{3}\times 470\times 10^{-12}}\approx 17.50\;\mathrm{kHz}$$

The zero obtained from the Beta1 curve is 350.03 kHz, and the pole is 18.43 kHz, both of which are very close to the calculated values.

#### 2.3.5. Stability Analysis

**Figure 14:**
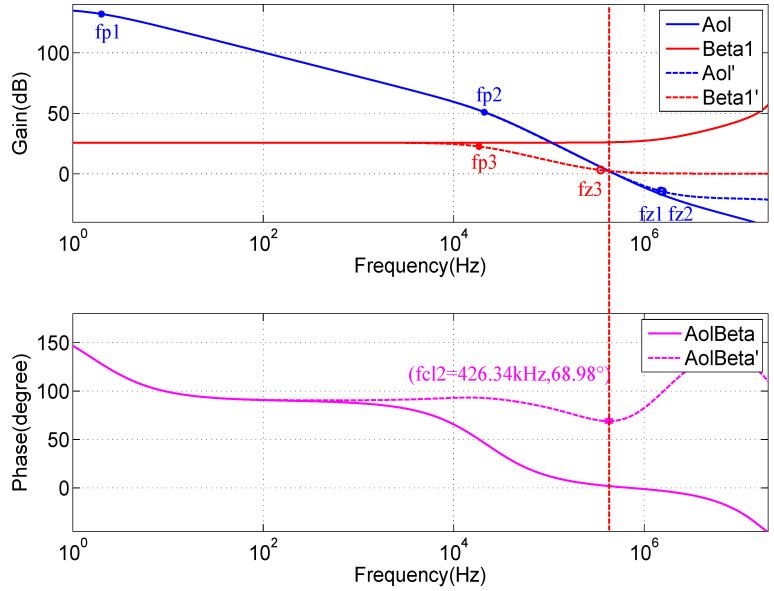
Comparison of Bode plots. The solid and dashed lines are the curves before and after compensation, respectively.

**Figure 15:**
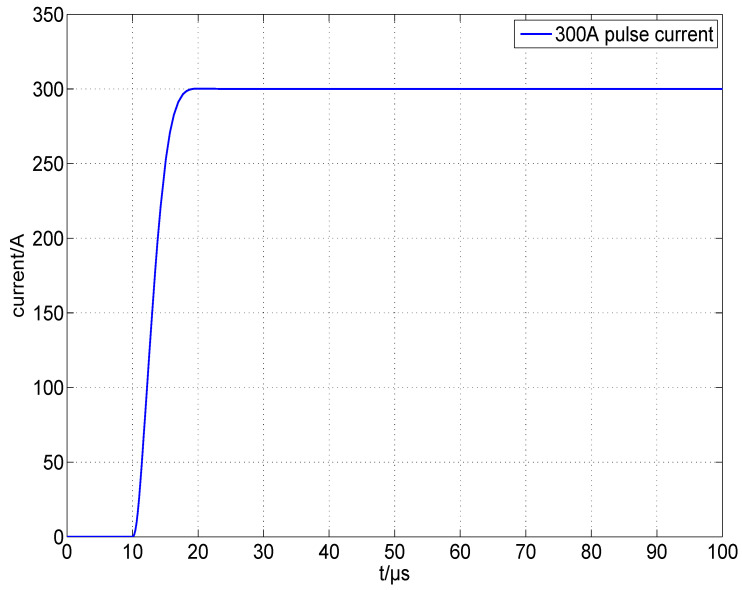
Small-signal transient simulation curve of the compensated circuit.

Figure 14 compares the Bode plots before and after compensation. The compensated open-loop gain curve $A_{ol}^{\prime }$ and closed-loop gain curve $Beta1^{\prime }$ intersect at $f_{CL2}=426.34$ kHz, where the rate of closure is slightly less than −20 dB/decade and the phase margin is $68.98^{\circ }$, indicating that the circuit is stable. Small-signal transient simulations performed on the circuit with an input voltage of 300 mV yield the output waveform shown in Figure 15, resulting in an output current of ∼300 A and a rising edge of ∼10 μs, with no significant overshoot. This set of component values can be used as a reference for circuit design and adjusted according to the test results of the actual circuit.

### 2.4. Implementation of the Load-Adaptive Driving Methods

#### 2.4.1. PID Control Theory

The core element of the load-adaptive driving method for the QCW LD driver is locking the drain-to-source voltage $V_{DS}$ of the voltage-controlled MOSFET to $V_{GS}-V_{TH}$. For engineering applications, instead of trying to study the transfer function of the system, the traditional digital PID control method can be employed since it does not require knowledge of the exact transfer function for feedback control, and our experiments demonstrated that this method works efficiently in our system. The following discusses the PID control method [31].

Equation (33) is a continuous expression of the PID algorithm:

(33) $$u\left(t\right)=K_{p}\left(e\left(t\right)+\frac{1}{T_{i}}\int_{0}^{t}e\left(t\right)dt+T_{d}\frac{de(t)}{dt}\right)$$

Equations (34) and (35) are discrete expressions of the PID algorithm:

(34) $$u_{x}=K_{p}\left(e_{x}+\frac{T}{T_{i}}\sum_{n=0}^{x}e_{n}+T_{d}\frac{e_{x}-e_{x-1}}{T}\right)$$

(35) $$u_{x-1}=K_{p}\left(e_{x-1}+\frac{T}{T_{i}}\sum_{n=0}^{x-1}e_{n}+T_{d}\frac{e_{x-1}-e_{x-2}}{T}\right)$$

The incremental PID algorithm, Equation (36), can be obtained by subtracting $u_{x-1}$ from $u_{x}$:

(36) $$\begin{array}{cc} \Delta u_{x} & =u_{x}-u_{x-1}=K_{p}\left(e_{x}-e_{x-1}+\frac{T}{T_{i}}e_{x}+T_{d}\frac{e_{x}-2e_{x-1}+e_{x-2}}{T}\right)\\ \; & =K_{p}\left(1+\frac{T}{T_{i}}+\frac{T_{d}}{T}\right)e_{x}-K_{p}\left(1+\frac{2T_{d}}{T}\right)e_{x-1}+K_{p}\frac{T_{d}}{T}e_{x-2}\\ \; & =Ae_{x}+Be_{x-1}+Ce_{x-2} \end{array}$$

The coefficients *A*, *B*, and *C* are defined by Equations (37)–(39), respectively:

(37) $$A=K_{p}\left(1+\frac{T}{T_{i}}+\frac{T_{d}}{T}\right)$$

(38) $$B=-K_{p}\left(1+\frac{2T_{d}}{T}\right)$$

(39) $$C=K_{p}\frac{T_{d}}{T}$$

where *x* is the sampling sequence number (i.e., *x* = 0, 1, 2, ……), $u_{x}$ is the output value of the PID at the sampling time *x*, $\Delta u_{x}$ is the output value of the incremental PID at the sampling time *x*, $e_{x}$ is the input error at sampling time *x*, $K_{p}$ is a proportional constant, $T_{i}$ is an integral constant, $T_{d}$ is a derivative constant, and *T* is the sampling time.

In this study, a microprocessor was used as the servo controller to implement the incremental PID control method. The sampling time was the same as the QCW LD driving period. To reduce the control frequency, we also set an error threshold such that the controller only worked if the input error exceeded the set value.

#### 2.4.2. Control Process of the Load-Adaptive Driving Method

**Figure 16:**
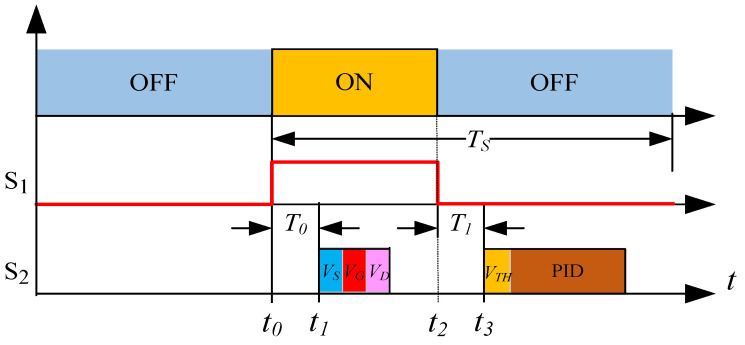
Control process diagram for the load-adaptive driving method.

According to the description of the load-adaptive driving method in Section 1.3.2, we need to lock the drain-to-source voltage $V_{DS}$ of the MOSFET to the set value $V_{DS\_SET}$ (see Equation (18) and (19)). Computing the PID input error requires sampling the values of $V_{D}$, $V_{G}$ and $V_{TH}$, which were obtained at different times. As shown in Figure 16, $S_{1}$ is the trigger pulse with period $T_{S}$, and $S_{2}$ represents the PID control sequence. The servo controller detects the rising edge of $S_{1}$ at time $t_{0}$ and samples $V_{S}$, $V_{G}$ and $V_{D}$ via ADCs at time $t_{1}$ after a delay of $T_{0}$. The falling edge of the pulse is detected at time $t_{2}$, and $V_{TH}$ is sampled at time $t_{3}$ after a delay of $T_{1}$. The error $V_{ERR}$ is then calculated per Equation (19), and $V_{DS}$ is adjusted to complete the PID control process. Of note, $V_{DS}$ is controlled indirectly by adjusting the output voltage $V_{CAP}$ of the linear voltage-controlled SMPS.

## 3. Experimental Result

### 3.1. Experiment Setup

**Figure 17:**
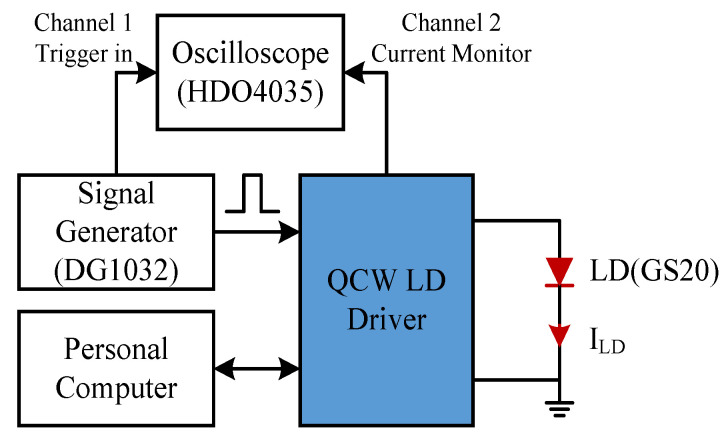
Diagram of the experimental setup.

Figure 17 shows the experimental setup used to test the QCW LD driver. The block diagram includes five components: an LD (FOCUSLIGHT GS20) as the driven load; a QCW LD driver, our equipment to be tested; a signal generator DS1032 (RIGOL 30 MHz, 200 MS/s), which provided the pulsed trigger signals; an oscilloscope HDO4035 (Lecroy 350 MHz, 2.5 GS/s) to measure the waveform of the triggered pulses and current pulses; and a personal computer to perform the parameter configuration and status monitoring for the driver.

### 3.2. Pulsed Constant-Current Source Test

**Figure 18:**
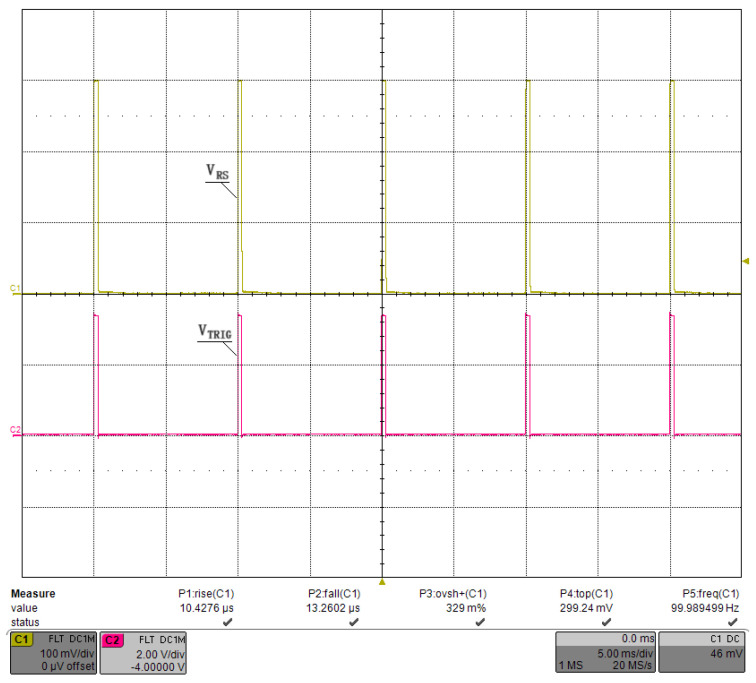
Waveforms of the current pulse sequence.

**Figure 19:**
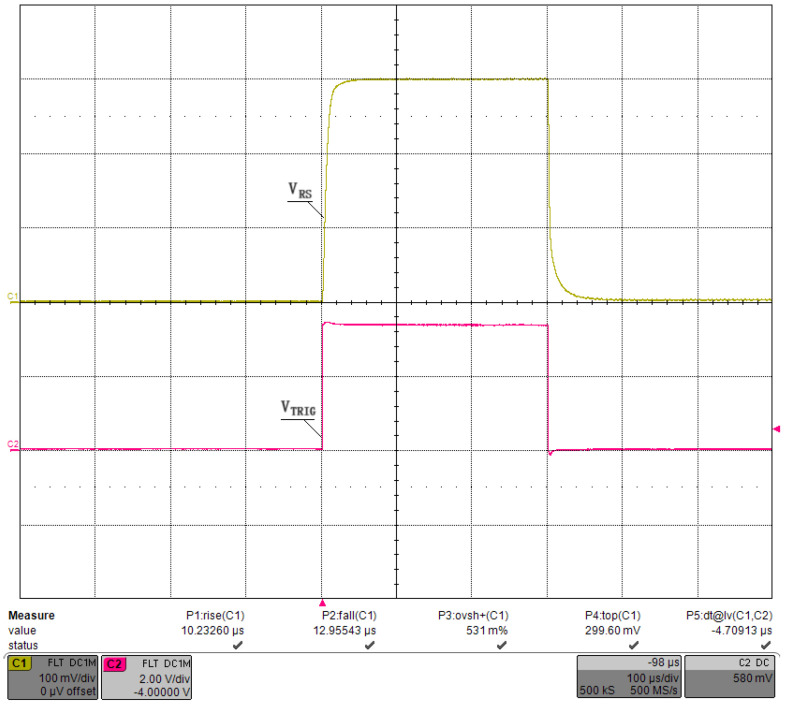
Waveforms the single-pulse current.

**Figure 20:**
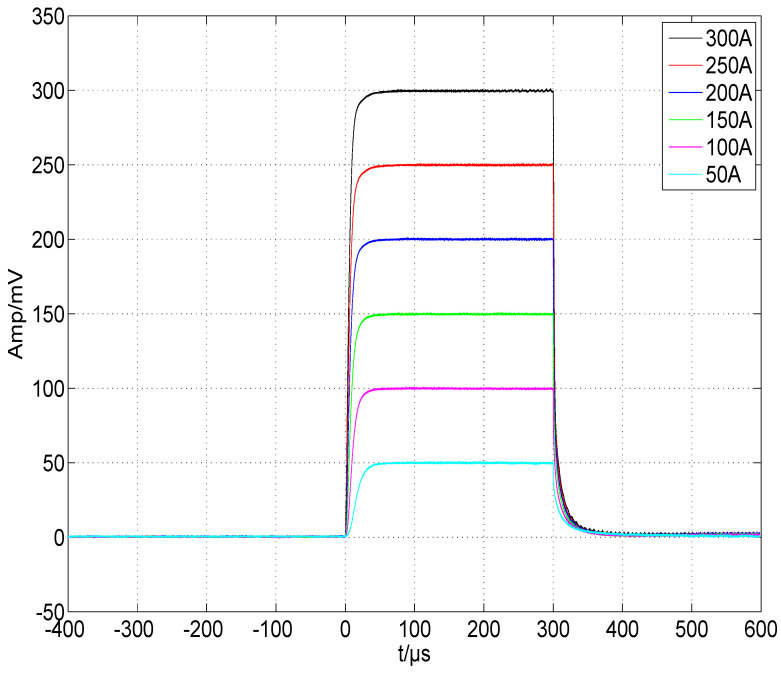
Current-pulse amplitude adjusted by a step of 50 A.

**Figure 21:**
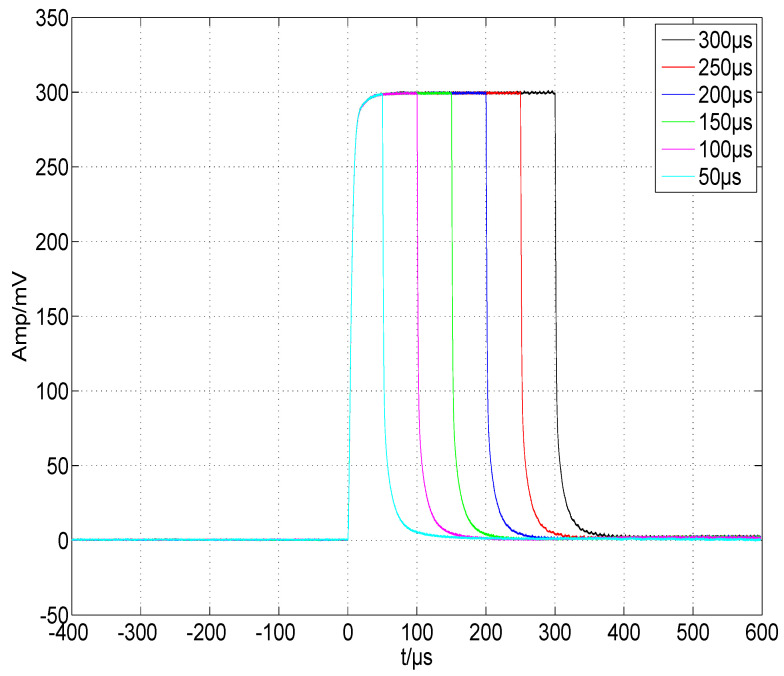
Current pulse width adjusted by a step of 50 μs.

We first debugged the pulsed constant-current source circuit shown in Figure 13, setting $V_{CAP}$ at a fixed value and retaining a sufficient margin so that $V_{DS}>V_{GS}-V_{TH}$ always held. The connection between the GS20 load under test and the driver was as short as possible to minimize the effect of parasitic inductance on the current pulse, and we used a low-inductance cable. Finally, we applied $R_{iso}=5.1\;\Omega$, $R_{f}=1\;k\Omega$, and $C_{f}=1000$ pF for the compensation. Figure 18 shows the waveform of the current pulse sequence, with a pulse width of ∼300 μs, a current of ∼300 A, and a repetition rate of 100 Hz. Channel 1 was the voltage waveform of $R_{S}$, named $V_{RS}$, and channel 2 was the waveform of the trigger pulse, named $V_{TRIG}$. Figure 19 shows the waveform of a single current pulse, with a rising time of 10.2 μs, a falling time of 13.0 μs, an overshoot of 0.53%, and a $V_{RS}$ of 299.6 mV, corresponding to a load current of 299.6 A. The current value was stepped by 50 A, and the results are plotted in Figure 20 using MATLAB, R2019. The pulse width was stepped by 50 μs, and the results are plotted in Figure 21. Finally, we sampled 1,000,000 values of the current pulse using an ADC inside the driver, and the statistical results are shown in Table 5. The above test results indicate that the shape and value of the current pulse can be properly controlled by the circuit and parameters designed in this study.

### 3.3. Load-Adaptive Driving Test

**Figure 22:**
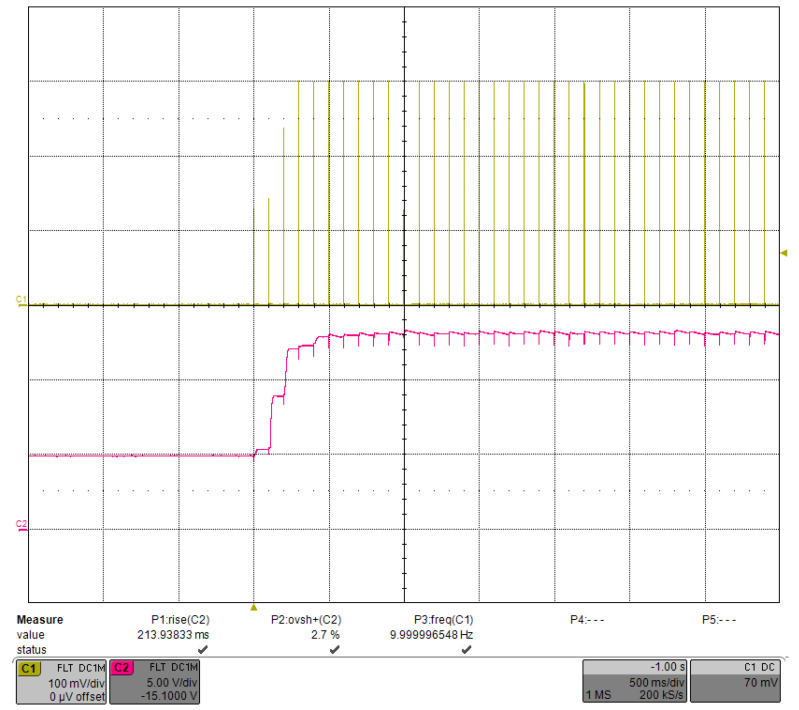
Step response under load-adaptive driving with a current of 300 A, a repetition rate of 10 Hz, and a pulse width of 300 μs.

Before performing this test, we set the bias voltage $V_{BIAS}$ such that the MOSFET would be in the micro-on state during idling, when the base voltage is approximately equal to the threshold voltage $V_{TH}$. We set the $V_{BIAS}$ to 0.1 mV, at which point $V_{TH}$ was measured to be 3.7 V. The operating range of $V_{CAP}$ was 5–15 V, with a low threshold of 5 V to obtain the correct $V_{TH}$ before the PID started and a high threshold of 15 V to protect the system. The classical Ziegler-Nichols (ZN) method was not fully applicable for tuning the PID parameters in our application, so we used manual tuning in the order of $K_{p}$, $K_{i}$ and $K_{d}$. The step response of the driver, shown in Figure 22, had a current of 300 A, a repetition rate of 10 Hz, and a pulse width of 300 μs. Channel 1 was the current pulse waveform with three transition pulses. Channel 2 was the $V_{CAP}$ waveform with a rising time of 213.9 ms and an overshoot of 2.7%. The glitches on the $V_{CAP}$ waveform were due to voltage drops caused by capacitor discharges.

**Figure 23:**
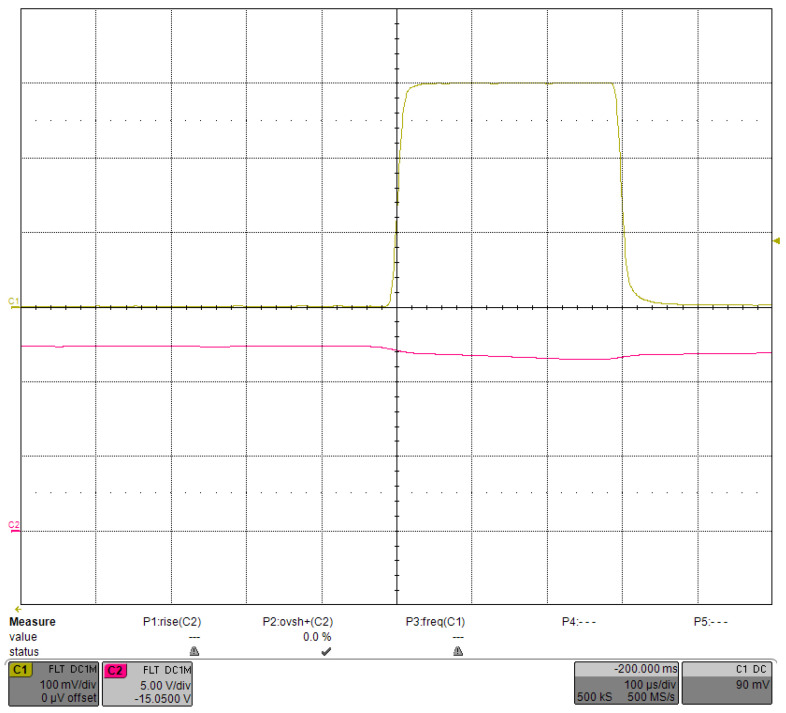
Single current pulse after the $V_{CAP}$ stabilization under load-adaptive driving.

**Figure 24:**
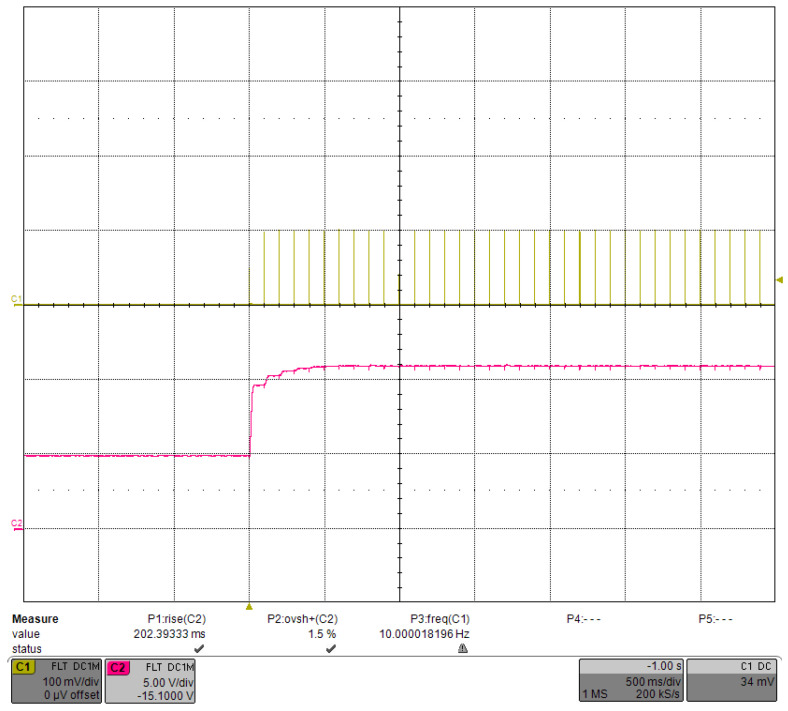
Step response under the load-adaptive driving when the current was reduced to 100 A.

The PID was able to control $V_{CAP}$ in a relatively stable voltage range. Figure 23 shows the waveform of a single current pulse after $V_{CAP}$ stabilization, and the current was very stable. The step response when the driving current was decreased to 100 A is shown in Figure 24. As the required $V_{CAP}$ voltage was reduced, the rising time was also reduced to 202.4 ms, with an overshoot of ∼1.5%. In addition, with the same PID parameters, the change in the output current had little effect on the transient waveform of $V_{CAP}$. We tried different PID parameters and showed that the number of transition pulses could no longer be reduced even if the rising time of $V_{CAP}$ was shortened.

**Figure 25:**
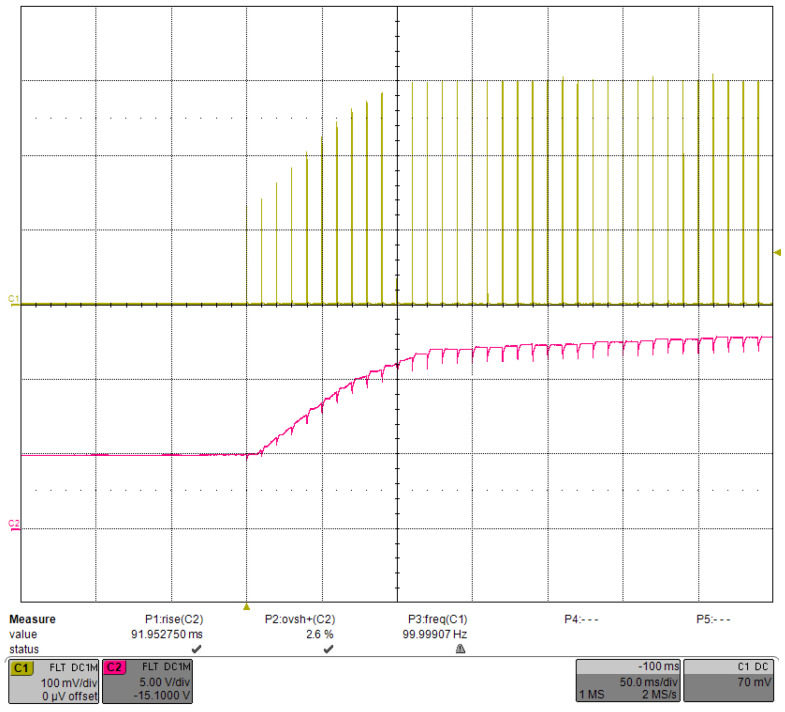
Step response under the load-adaptive driving with the PID parameters unchanged but the repetition rate increased to 100 Hz.

**Figure 26:**
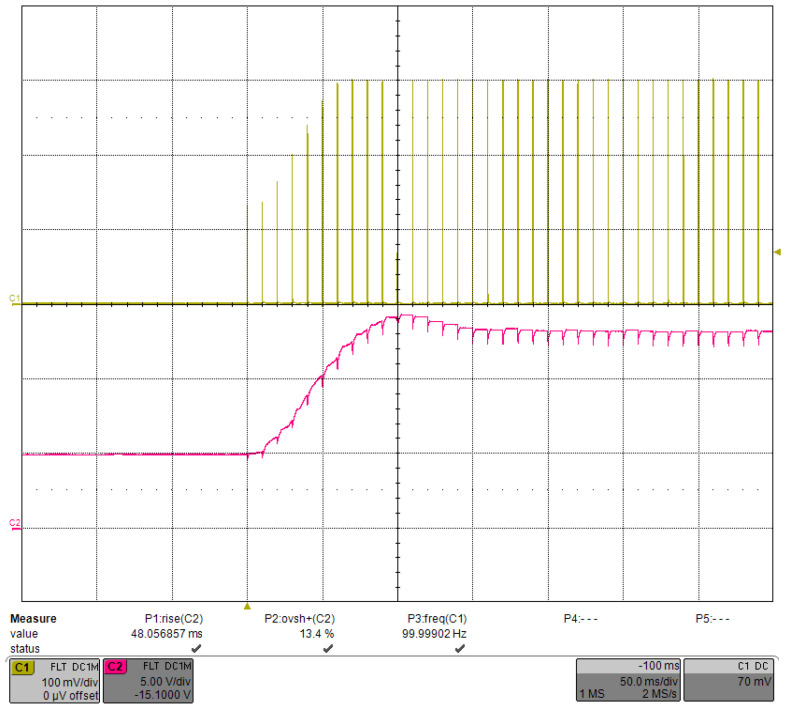
Step response under load-adaptive driving with a current of 300 A, a repetition rate of 100 Hz, and a pulse width of 300 μs.

With the same PID parameters, the repetition rate was increased to 100 Hz, and the corresponding sampling time was reduced to 10 ms. The step response is shown in Figure 25. The $V_{CAP}$ rising time was 92.0 ms, and the overshoot was 2.6%, which was still well-controlled. However, the number of transition pulses was increased to 10, which was excessive. After we optimized the PID parameters, the rising time of $V_{CAP}$ decreased to 48.1 ms, the overshoot increased to 13.4%, and the number of transition pulses decreased to six, as shown in Figure 26. Although the $V_{CAP}$ overshoot increased, it accelerated the stabilization time. In practice, the same PID parameters were used for the driver since a few more transition pulses would have little effect on the system.

### 3.4. Power Efficiency Test

Power efficiency is one of the most critical characteristics of an LD driver. We compared the efficiency of the pulsed constant-current source circuit experimentally with and without the load-adaptive driving method. Since the SMPS is a mature commercial module, we excluded it from the efficiency evaluation and considered it equivalent under both tests. The power efficiency was calculated according to Equation (40):

(40) $$\eta =\frac{V_{LD}\times I_{D}}{\bar{V_{CAP}}\times I_{D}}=\frac{V_{LD}}{\bar{V_{CAP}}}$$

where $\bar{V_{CAP}}$ is the average voltage of the driver during operations and $V_{LD}$ is the voltage of the *LD* under the test.

**Figure 27:**
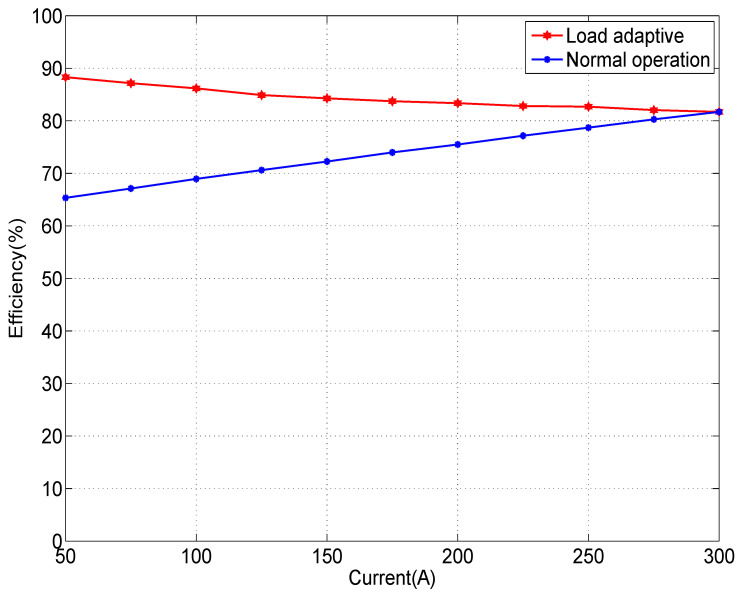
Comparison of the power efficiency in the normal operation mode and the load-adaptive mode.

Based on Equation (7), when the $V_{LD}$ is greater than the threshold voltage, it is approximately proportional to its current because of the effect of the equivalent resistance $R_{LD}$. Therefore, in the normal operation mode, since $V_{CAP}$ is fixed, the *LD* voltage decreases as $I_{D}$ decreases, and the efficiency drops. For comparison, we carefully set $V_{CAP}$ such that $V_{DS}\approx V_{GS}-V_{TH}$ at $I_{D}=300$ A. The measured efficiency is shown as the blue curve in Figure 27, which aligns well with Equation (7).

When the driver operated in a load-adaptive mode, the servo locked $V_{DS}$ to $V_{GS}-V_{TH}$. When the load current $I_{D}$ decreased, $V_{DS}$ decreased along with the required $V_{CAP}$ accordingly, which was consistent with the characteristic curve of the MOSFET, as shown in Figure 3. As a result, the efficiency of the driver increased with the load shown by the red curve in Figure 27.

Of note, we could not set a small $V_{CAP}$ at $I_{D}=50$ A in normal operation mode for this experiment as the $V_{DS}$ would decrease with $I_{D}$, and the MOSFET would eventually be unable to output a sufficient current. Further improvement in the efficiency of the driver is possible in the load-adaptive mode as long as *a* in Equation (18) is reduced, but $V_{DS}$ requires a sufficient margin to ensure stability.

### 3.5. Test of Driving a QCW Laser

**Figure 28:**
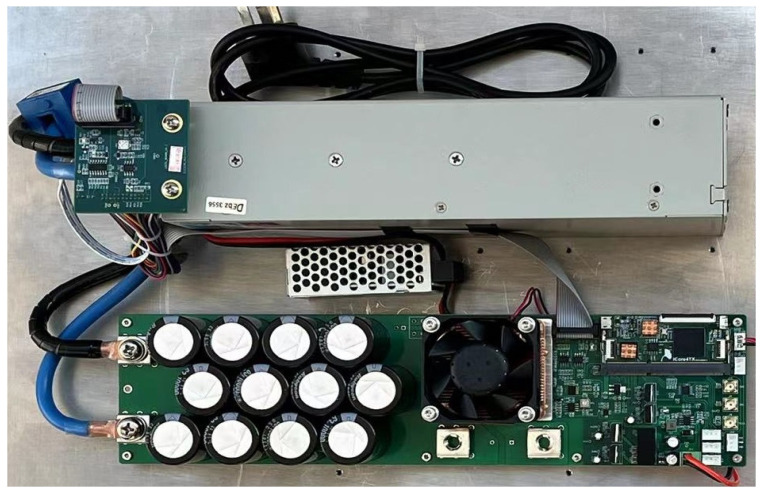
Photograph of the prototype QCW LD driver.

**Figure 29:**
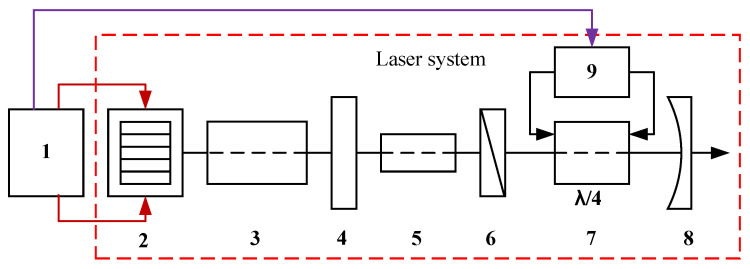
Optical arrangement of the tested QCW laser: (1) QCW LD driver; (2) LD stack GS20; (3) pump coupler; (4) rear mirror; (5) Nd:YAG; (6) polarizer; (7) Pockels cell; (8) output mirror; (9) Pockels cell driver.

**Figure 30:**
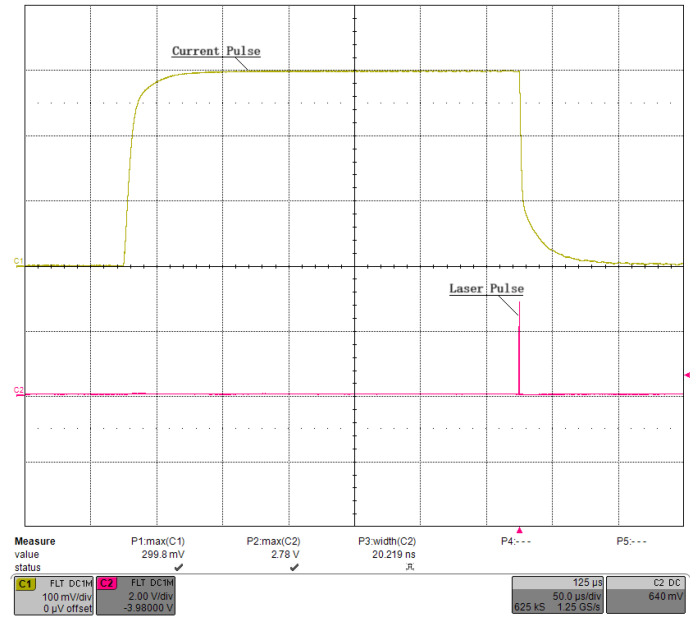
Laser pulse generated by the tested QCW laser.

**Figure 31:**
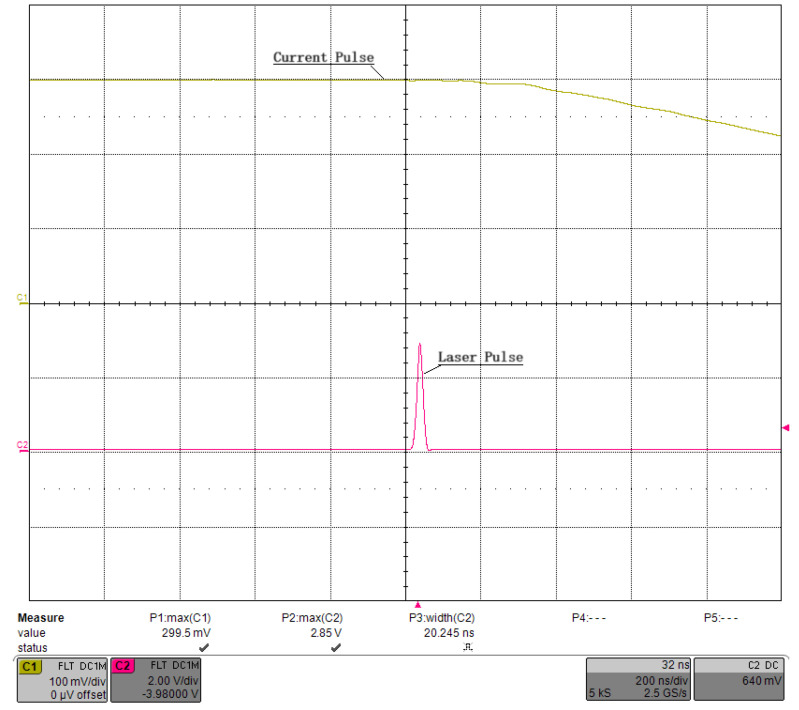
Zoomed-in image of the laser pulse shown in Figure 30.

Figure 28 shows a photograph of the developed QCW LD driver, which consists of three components: a commercial SMPS with linearly adjustable voltage, a pulsed constant-current source circuit board with load-adaptive driving, and a low-power AC/DC module to power the board. We verified the functionality of this driver in a home-built laser system with the optical arrangement shown in Figure 29. The QCW LD driver operated with a current of 300 A, a pulse width of 300 μs, and a repetition rate of 100 Hz and drove the LD stack GS20 to generate an 808 nm pump light, which was injected into the laser resonator via a pump coupler. The Pockels cell driver used quarter-wave “off-type” voltage to keep the resonator closed, and it switched down to zero at the falling edge of the current pulse. At this point, the Q-factor of the laser resonator dropped instantaneously, and a high-energy 1064 nm laser pulse was immediately emitted. In Figure 30, channel 1 is the waveform of the current pulse, and channel 2 is the optical pulse received by the photodiode. Figure 31 shows a zoomed-in version of Figure 30. The laser pulse width was 20.2 ns, the average power measured by the optical power meter was 3.93 W, and the laser worked well.

## 4. Conclusions

In this study, we designed a pulsed constant-current source based on a voltage-to-current converter circuit of op-amps and a MOSFET. The stability of the circuit was addressed by adding compensation elements and completing circuit simulations. Based on the MOSFET’s properties, we propose an efficient and load-adaptive driving method for the QCW LD. Compared with a traditional driver, this driver has not only the advantages of high precision and high stability but also the ability to adjust the operating voltage automatically according to the load characteristics and load changes, which is very suitable for the application of a power- and stability-sensitive QCW laser. The experimental results showed that the driver can generate a pulsed constant current with a repetition rate of 100 Hz, a current of 300 A, and a pulse width of 300 μs. The efficiency of the pulsed constant-current source was consistently above 80% when the load current varied from 50 to 300 A, which we demonstrated with our home-built laser system. We designed only a prototype of the driver for the time being, but the long-term stability and protection mechanisms that are equally important for a commercial product are not discussed in this paper, which is recommended for further study.

## Figures and Tables

**Table 1:** Design specifications.

Parameters	Values
Operating current	50–300 A
Pulse width	50–300 μs
Repetition rate	10–100 Hz
Load voltage	<10 V

**Table 2:** Parameters of the IXTH360N055TT2.

Parameters	Values
Drain-to-source breakdown voltage	55 V
Drain-to-source current capability	360 A
Gate-to-source voltage	±20 V
Gate threshold voltage	2.0-4.0 V
Drain-to-source on-resistance	∼2.4 mΩ

**Table 3:** OPA2197 parameters.

Parameters	Values
Gain bandwidth	10 MHz
Open-loop gain	134 dB
Differential input impedance	100 MΩ
Open-loop output impedance	375 Ω
Input offset voltage	±25 μV
Input offset current	±5 pA
Supply voltage	±18 V

**Table 4:** Main parameters of the AE-1500-24 SMPS.

Parameters	Values
Voltage range	0–24 V
Current range	0–62.5 A
Rated power	1500 W
Efficiency (max.)	92%

**Table 5:** Statistical results for the 1000,000 current pulses.

Minimum Value (A)	Maximum Value (A)	Mean Value (A)	Standard Deviation (A)
299.1	300.7	299.9	0.2

## Data Availability

Data are contained within the article.
